# Two new species of *Urothoe* (Crustacea, Amphipoda, Gammaridea) from the East Johor Islands Archipelago, Malaysia

**DOI:** 10.3897/zookeys.87.817

**Published:** 2011-03-24

**Authors:** B.A.R. Azman, C.W.H. Melvin

**Affiliations:** Marine Science Programme, School of Environmental & Natural Resource Sciences, Faculty of Science and Technology, Universiti Kebangsaan Malaysia, 43600 UKM Bangi, Selangor, Malaysia

**Keywords:** Crustacea, Amphipoda, Urothoidae, *Urothoe tinggiensis*, *Urothoe sibuensis*, East Johor Islands Archipelago

## Abstract

Two new species of urothoid amphipods from Pulau Sibu and Pulau Tinggi, Johor are described and illustrated. The specimens of *Urothoe sibuensis* new species were collected by vertical haul plankton net and is distinctively different from other existing *Urothoe* species by these combination of special characters; similar gnathopods 1–2 with short and stout propodus expanded into poorly defined palms; large eyes and epimeron 3 smooth. *Urothoe tinggiensis* new species as collected using an airlift suction sampler at seagrass area is characterized by its different gnathopodal configuration with setose dactylus of 5th pereopod; eyes minute; carpus is wider than merus in the 5th pereopod; subquadrate coxa 4; merus and carpus of pereopods 6–7 are linear.

## Introduction

The genus *Urothoe* Dana, 1852 has been known to be cosmopolitan and is one of the most diverse genus of the family Urothoidae Bousfield, 1978. They are easily recognized by their small sized body and furnished with various fossorial adaptations in the form of extremely setose appendages (Barnard and Drummond 1982). They are also known to be very diverse in shallow habitats and act as an important component of the burrowing fauna of intertidal and shallow subtidal areas but scarce in the deep sea (Barnard and Karaman 1991).

According to Bousfield’s (1978) classification, the family Urothoidae is characterized by a 7-segmented pereopod 5 with merus and propodus stoutly expanded. He included nine genera, namely *Urothoe* Dana, 1852 as type genus; *Carangolia* Barnard, 1961; *Cunicus* Griffiths, 1974; *Haustoriella* K.H. Barnard, 1931; *Phoxocephalopsis* Schellenberg, 1931; *Urothopsis* Ledoyer, 1969; *Urohaustorius* Sheard, 1936; *Urothoides* Stebbing, 1891; and *Zobracho* Barnard, 1961. However in 1982, Barnard and Drummond made a revision on the family and only retained *Urothoides*, *Cunicus*, *Carangolia* and *Urothoe* as members of the Urothoidae that possess the large ventral cheek on the head, the styliform-lanceolate and setose rami of uropods 1 & 2. Later, an additional two genera (*Urothopsis* and *Pseudourothoe*) were added bringing it to a total of six genera under the family Urothoidae (Barnard and Karaman 1991).

Up till now, only one species, *Urothoe gelasina* Imbach, 1969 was recorded in Malaysian waters by Azman (2007), which was previously recorded in the Bay of Nhatrang, Vietnam. Taxonomic studies on Amphipoda in this region particularly in Malaysian waters is still sparse, nevertheless there is an increasing attempt to improve the knowledge and several contributions have been recently published such as Müller (1993), (Othman and Morino (1996, 2006), Othman and Azman (2007), Azman (2007), and Lim et al. (2010). In this paper we describe two new species from this region: *Urothoe sibuensis* new species and *Urothoe tinggiensis* new species.

## Material and methods

**Figure 1.**
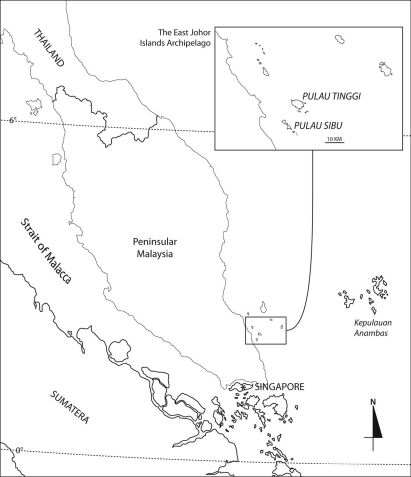
Pulau Tinggi and Pulau Sibu of East Johor Islands Archipelago, Malaysia.

The amphipods in this study were obtained from Pulau Tinggi for *Urothoe tinggiensis* and Pulau Sibu for *Urothoe sibuensis*; both from the waters of Johor, southeast coast of Peninsular Malaysia (Figure 1). Airlift suction sampler and plankton net of 100 µm mesh size were used in the collection of the specimens. All materials are lodged in the Universiti Kebangsaan Malaysia Muzium Zoologi (UKMMZ). The following abbreviations are used on the plates: A, antenna; G, gnathopod; HD, head; LL, lower lip; MD, mandible; MX, maxilla; MP, maxilliped; P, pereopod; PL, pleopod; EP, epimeron; T, telson; U; uropod; UR, urosome; UL, upper lip; ♂, male; ♀, female.

## Results

### Systematics

**Urothoidae Bousfield, 1978**

#### Urothoe

Dana, 1852

##### Type species.

*Urothoe irrostratus* Dana, 1853, original designation.

##### Diagnosis.

Molar process moderately to strongly developed. Gnathopods weak, subequal, variously subchelate. Pereopod 5 distinctly 7-segmented. Pereopods 6 and 7 subsimilar in form and size, strongly fossorial. Pereopod 7 not of phoxocephalid form, basis not shield-like. Abdomen not sharply narrowing or flexed at urosome. Uropod 3 rami subequal; long and slender. Telson deeply cleft.

##### Species composition.

*Urothoe* contains fourty-five species: *Urothoe abbreviata* Sars, 1879; *Urothoe atlantica* Bellan-Santini & Menioui, 2004; *Urothoe bairdii* Bate, 1862; *Urothoe brevicornis* Bate, 1862; *Urothoe carda* Imbach, 1969; *Urothoe chosani* Hirayama, 1992; *Urothoe corsica* Bellan-Santini, 1965; *Urothoe convexa* Kim & Kim, 1992; *Urothoe coxalis* Griffiths, 1974; *Urothoe cuspis* Imbach 1969; *Urothoe dentata* Schellenberg, 1925; *Urothoe denticulata* Gurjanova, 1951; *Urothoe elegans* Bate, 1857; *Urothoe elizae* Cooper & Fincham, 1974; *Urothoe falcata* Schellenberg, 1931; *Urothoe gelasina* Imbach, 1969; *Urothoe gelasina ambigua* Hirayama, 1988; *Urothoe grimaldii* Chevreux, 1895; *Urothoe grimaldii japonica* Hirayama, 1988; *Urothoe irrostrata* Dana, 1853; *Urothoe hesperiae* Conradi, Lopez-Gonzalez & Bellan-Santini 1995; *Urothoe intermedia* Bellan-Santini & Ruffo, 1986; *Urothoe latifrons* Ren 1991; *Urothoe leone* Reid, 1951; *Urothoe marina* (Bate, 1857); *Urothoe marionis* Bellan-Santini & Ledoyer, 1986; *Urothoe oniscoides* (K.H. Barnard, 1932); *Urothoe orientalis* Gurjanova, 1938; *Urothoe pestai* Spandl, 1923; *Urothoe pinnata* K.H. Barnard, 1955; *Urothoe platydactyla* Rabindranath, 1971; *Urothoe platypoda* Griffiths, 1974; *Urothoe poseidonis* Reibisch, 1905; *Urothoe poucheti* Chevreux, 1888; *Urothoe pulchella* (Costa, 1853); *Urothoe rotundifrons* J.L. Barnard, 1962; *Urothoe ruber* Giles, 1888; *Urothoe serrulidactyla* K.H. Barnard, 1955; *Urothoe sibuensis* sp. n.; *Urothoe spinidigitus* Walker, 1904; *Urothoe tinggiensis* sp. n.; *Urothoe tumorosa* Griffiths, 1974; *Urothoe varvarini* Gurjanova, 1953; *Urothoe vemae* J.L. Barnard, 1962; *Urothoe wellingtonensis* Cooper, 1974.

##### Remarks.

Dana established the genus *Urothoe* in 1852 with the description of *Urothoe irrostrata*, which was originally classified under the family Haustoriidae. However, in 1978, Bousfield did a thorough revision on the phylogenetic classification of the genus *Urothoe* and it was decided that the genus *Urothoe* was to be moved to the Urothoidae family. The genus *Urothoe* is closely related to *Urothoides* Stebbing, 1891, but differs in not presenting the phoxocephalid-like protruding shield of basis on pereopod 7 (Barnard and Drummond 1979). There are about 45 species of *Urothoe* recorded to date (including the two present species), ten species were found from the South China Sea while the others were recorded from the waters of northeast Atlantic, Mediterranean Sea, New Zealand, Japan, Korea, South Africa, Australia and the North East Atlantic.

##### Urothoe sibuensis sp. n.

urn:lsid:zoobank.org:act:05F15CE6-9C13-4481-B623-A7D9F2F1A41D

Figs 2A [Fig F3] [Fig F4] [Fig F5] –2E 

###### Type material.

Holotype, male, 2.9 mm, UKMMZ-1394, seagrass area (*Halophila ovalis*, *Halodule uninervis*, *Cymadocea serrulata*, *Halophila spinulosa*) of Pulau Sibu, Johor, 2°13'55"N, 104°3'14"E, vertical haul plankton net (100 µm), 8 m, B.A.R. Azman, Melvin, C.W.H., Yoshida, T., 16 October 2008 (UKM I.D. 9047). Paratypes: 8 males, UKMMZ-1396, same station data.

###### Type locality.

Pulau Sibu, Johor, Malaysia, South China Sea.

###### Etymology.

This species is named after the type locality, Pulau Sibu, Johor.

**Figure 2a.**
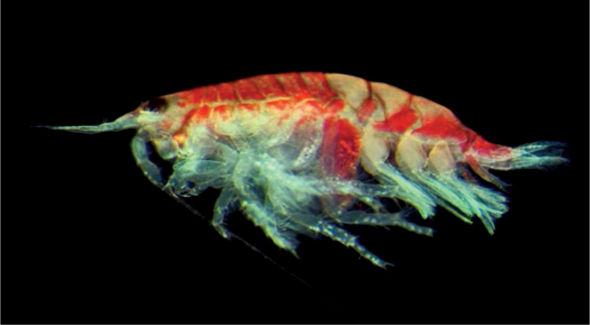
*Urothoe sibuensis* sp. n., holotype, male, (UKMMZ-1394), 2.9 mm. Pulau Sibu, Johor, South China Sea.

###### Description.

Based on holotype male, 2.9 mm, UKMMZ-1394.

####### Head

subequal in length to pereonite 1 – 3 combined, rostrum absent, lateroventral cephalic lobe less pronounced. *Eyes* present, well-developed, elliptic shape and large, lateral cephalic lobe of eye advanced slightly forward. *Antenna 1* shorter than antenna 2; peduncular article 1 slightly longer than article 2, provided with a band of setae on anterior margin; main flagellum with about 7 articles, distinctively shorter than peduncle; accessory flagellum biarticulate. *Antenna 2* about as long as body length; peduncle article 4 about twice as broad as long; gland cone absent; peduncular article 4 with robust setae along anterior margin; peduncular article 4 about as long as peduncular article 5; calceoli present; flagellum with about 30 articles. *Upper lip* semicircular. *Lower lip* inner plate large, shoulders broad, mandibular process significantly winged outward. *Maxilla 1* inner plate with one plumose setae apically; outer plate biarticulate, one setae and one plumose setae apically; palp with10 robust setae apically. *Maxilla 2* inner and outer plate covered with pinnate and simple setae apically. *Mandible* with molar semicircular and not well-developed; incisors smooth. *Maxilliped* inner plate elongated, bearing two blunt robust setae; outer plate suboval, with about 8 robust setae; palp 4-articulate, first article subquadrate, second article the widest, strongly setose on the inner margin, third article subtriangular, with several long setae; fourth article small and slender.

####### Pereon

*Gnathopod 1* coxa subrectangular, narrow; basis elongate, posterior margin with several long and short setae; ischium subtriangular with several short setae along posterior margin; merus semicylindrical; carpus longer than propodus, about twice as broad as long, posterior margin densely setose; propodus slightly expanded distally; palm with several setae; dactylus large with setae at base. *Gnathopod 2* coxa expanded ventrally; basis elongate with several long setae along posterior margin; ischium subtriangular with several long setae along posterior margin; merus subtriangular with rarely long and short setae present; carpus broad, longer than propodus, propodus subtriangular; dactylus similar to the dactylus of gnathopod 1.

*Pereopod 3 – 4* almost homopodous except pereopod 3 slightly broader than pereopod 4. *Pereopod 3* coxa slightly expanding ventrally, basis elongate with few long setae at distal end of posterior margin; ischium broader than long with few long setae; merus twice as long as carpus, posterior margin with dense setae and several plumose setae; carpus subquadrate, several setae, plumose setae and robust setae present; propodus narrow, armed with several robust setae along posterior margin; dactylus nodulate. *Pereopod 4* coxa subtriangular; basis elongate, few long setae with a plumose setae; ischium rarely setose; merus elongate, setose at posterior margin; carpus covered with several robust setae, setae and plumose setae; propodus narrow, with several robust setae; dactylus nodulate. *Pereopod 5* coxa bilobial; basis expanding backward, several setae in the notch of posterior margin, several plumose setae situated distally; ischium wider than long; merus wider than long provided with transverse rows of robust setae medially and distally, several long plumose setae distally; carpus subequal of length and width, provided with traverse rows of spines medially and distally, several long plumose setae distally; propodus provided with 3 traverse rows of robust setae ventrally and 2 transverse rows of robust setae posteriorly, several long plumose setae medially; dactylus nodulate. *Pereopods 6 – 7* slender from basis to propodus except for dactylus of pereopod 6 nodulate whereas dactylus of pereopod 7 slender and smooth, basis rounded posteriorly.

####### Pleon

*Pleopods 1–3* peduncle distinctly longer than broad; biramous, multiarticulate. *Uropod 1* peduncle with two rows of robust setae; rami subequal in length with long robust setae medially. *Uropod 2* peduncle provided with several robust setae; rami subequal in length, outer ramus with a long robust setae medially and a distal setae. *Uropod 3* well developed; both rami with long plumose setae; outer ramus 2 biarticulate; inner ramus shorter than outer ramus. *Telson* about 5/6 cleft; both lobes with robust setae apically and few setae medially and distally.

**Figure 2b. F3:**
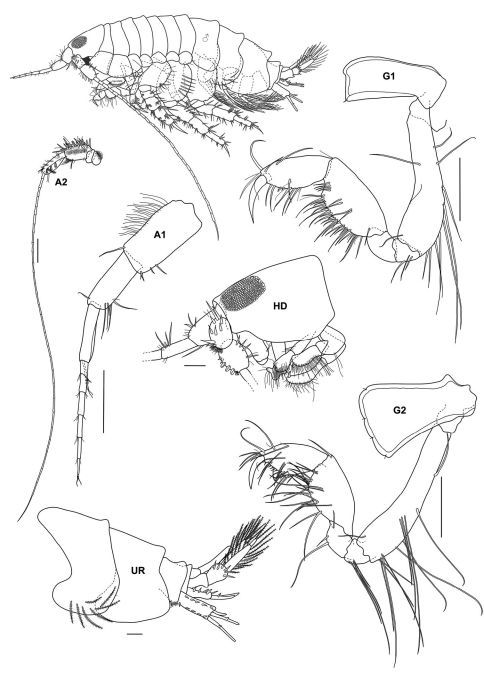
*Urothoe sibuensis* sp. n., holotype, male, (UKMMZ-1394), 2.9 mm. Pulau Sibu, Johor, South China Sea. All scales represent 0.1 mm.

**Figure 2c. F4:**
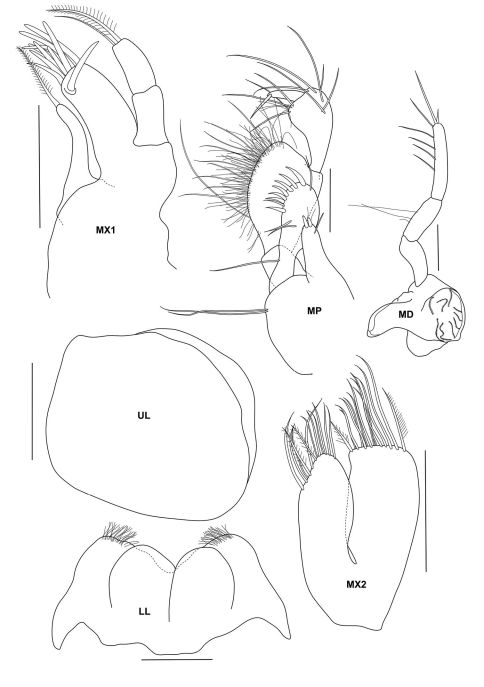
*Urothoe sibuensis* sp. n., holotype, male, (UKMMZ-1394), 2.9 mm. Pulau Sibu, Johor, South China Sea. All scales represent 0.05 mm.

**Figure 2d. F5:**
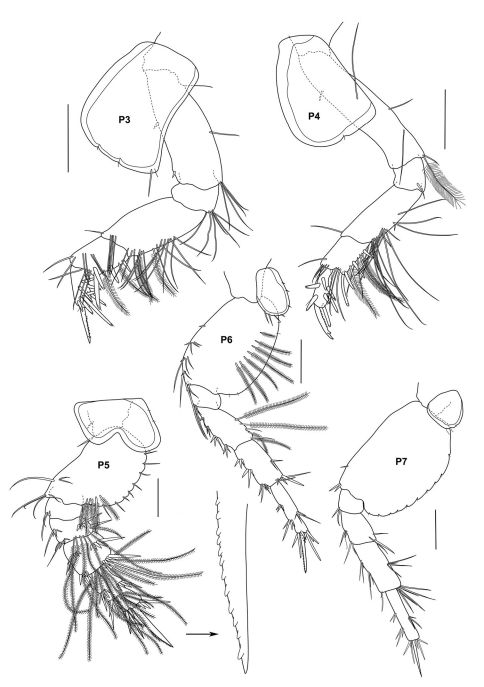
*Urothoe sibuensis* sp. n., holotype, male, (UKMMZ-1394), 2.9 mm. Pulau Sibu, Johor, South China Sea. All scales represent 0.1 mm.

**Figure 2e.**
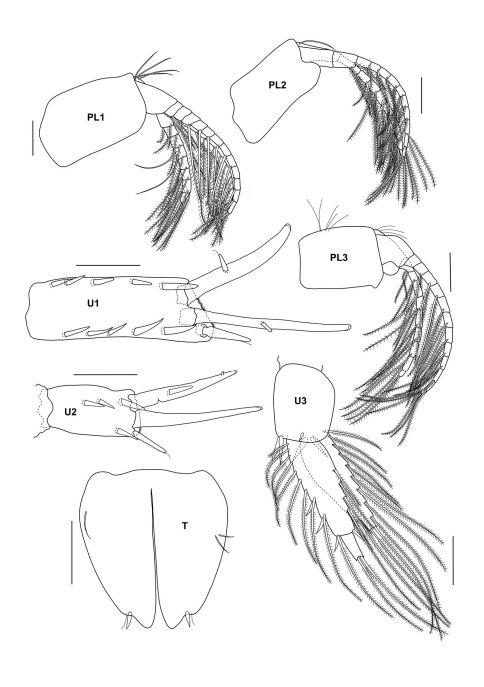
*Urothoe sibuensis* sp. n., holotype, male, (UKMMZ-1394), 2.9 mm. Pulau Sibu, Johor, South China Sea. All scales represent 0.05 mm.

###### Remarks.

*Urothoe sibuensis* is very closely related to the ‘elegans’ group, defined by J.L. Barnard (1962), which included twenty other *Urothoe* species. This group of species is characterized by having similar gnathopods 1 – 2 with short and stout propodus expanded into poorly defined palms. Despite of that, detectable distinctions of morphological characters are found between the present species and the members of this group. As such, *Urothoe sibuensis* differs in not having defining robust setae in propodus of gnathopods 1 – 2 (defining robust setae present in *Urothoe gelasina*, *Urothoe gelasina ambigua*, *Urothoe poucheti*, *Urothoe varvarini*), the antenna 2 significantly longer than antenna 1 (antenna subequal in length in *Urothoe bairdii*, *Urothoe brevicornis*, *Urothoe hesperiae*, *Urothoe intermedia*, *Urothoe marina*), epimeron 3 smooth (epimeron 3 having distinct tooth in *Urothoe chosani*, *Urothoe dentata*, *Urothoe denticulata*, *Urothoe marionis*), presence of eyes (absence of eyes in *Urothoe abbreviata*, *Urothoe latifrons*, *Urothoe vemae*), merus and carpus of pereopod 5 are both subequal in width (merus is wider than carpus of pereopod 5 in *Urothoe pulchella*, *Urothoe spinidigitus*), epimeron 2 having several plumose setae (epimeron 2 naked in *Urothoe elegans*) and a less pronounced cephalic lobe (lateroventral cephalic corner of *Urothoe corsica* produced and upturned).

The present species is especially close to *Urothoe spinidigitus* Walker, 1904. It differs from *Urothoe spinidigitus* by the absence of rostrum in the present species (small rostrum present in *Urothoe spinidigitus*) and the absence of defining palmar spines in both gnathopods. The dactylus of pereopod 5 in Walker’s species bears 4 short and 4 long robust setae whereas *Urothoe sibuensis* possesses a nodulate dactylus in its pereopod 5. The subequal merus and carpus of pereopod 5 in the present species clearly distinguish it from *Urothoe spinidigitus* that has carpus twice as broad as merus.

###### Distribution.

*Malaysia*. Johor, Pulau Sibu.

##### Urothoe tinggiensis sp. n.

urn:lsid:zoobank.org:act:806054F6-4A04-4F49-B13C-1A80B65A11C7

Figs 3A [Fig F8] [Fig F9] [Fig F10] 3E 

###### Type material.

Holotype, female, 2.0 mm, UKMMZ-1399, muddy-sand substrate of Pulau Tinggi, 2°16'51.70"N, 104°7'15.96"E, airlift suction sampler, 9 m, B.A.R. Azman, C.W.H. Melvin, J.H.C. Lim, 19 July 2007, UKM I.D. 8632. Paratype: 4 females UKMMZ-1401, same station data.

**Figure 3a.**
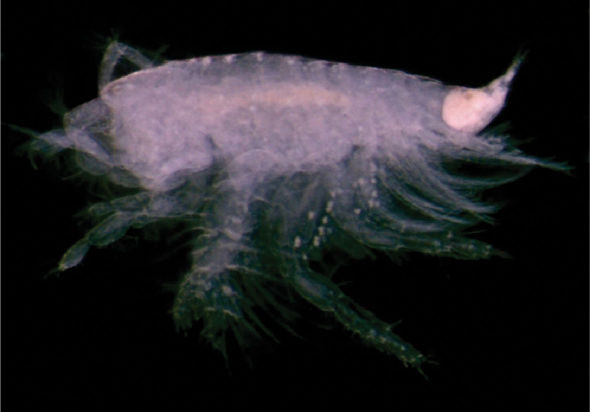
*Urothoe tinggiensis* sp. n., holotype, female, (UKMMZ-1399), 2.0 mm. Pulau Tinggi, Johor, South China Sea.

###### Type locality.

Pulau Tinggi, Johor, Malaysia, South China Sea

###### Etymology.

Named after the type locality, Pulau Tinggi in East Johor, Malaysia.

###### Description:

Based on holotype female, 2.0 mm, UKMMZ-1399.

####### Head

lateroventral cephalic lobes rounded and very pronounced, rostrum absent. *Eyes* minute, circular. *Antenna 1* peduncular articles 1 – 2 provided with setae along the ventral margin; accessory flagellum triarticulate; main flagellum distinctly shorter than peduncle, about as long as peduncle article 3, 5 articulate. *Antenna 2* subequal to antenna 1; flagellum biarticulae; first article with several robust setae and long setae all over; second article with several robust setae and long setae. *Upper lip* semicircular. *Lower lip* inner plate large, shoulders broad, mandibular process prominently winged outward. *Maxilla 1* inner plate with pinnate seta apically; outer plate with two setae, one pinnate and a seta; palp densely filled with robust setae apically. *Maxilla 2* inner and outer plates furnished with pinnate and simple setae. *Mandible* molar rounded and well-developed; incisor smooth; palp strong; first article shorter than second article; second and third article subequal in length; third article with several robust setae distally. *Maxilliped* inner plate elongate with about 2 blunt robust setae; outer plate suboval with several spines and setae; palp 4-articulate, second article wide and densely setose on inner margin, third article subtrianglular with several long setae, fourth article subtriangular and small.

####### Pereon

*Gnathopod 1* coxa narrow, subrectangular; basis elongate with several long setae along the posterior margin; carpus broad furnished with several setae at posterior margin, about as broad as long; propodus wide with several long setae situated anterodistally; dactylus normal. *Gnathopod 2* coxa narrow, subrectangular; basis elongate with several long setae along the posterior margin; carpus suboval in shape; propodus wide provided with several long setae at anterodistal angle; dactylus falcate, slightly extending palm when closed.

*Pereopod 3 – 4* homopodous, coxa subquadrate; basis elongate provided with few long setae; carpus twice as long as merus; propodus narrow; dactylus nodulate. *Pereopod 5* coxa small, bilobate; basis semicircular, few plumose setae along posterior margin; ischium wider than long; merus wider than long, provided with two transverse rows of robust setae and a row of plumose setae; carpus twice as long as wide, 4 transverse rows of robust setae, one traverse row of plumose setae; propodus longer than wide, provided with 3 traverse rows of robust setae; dactylus large, with 3–4 robust setae along anterior margin. *Pereopod 6* coxa subquadrate; basis wide, oval shape, several plumose setae along posterior margin; merus longer than wide; carpus longer than wide, robust setae along anterior margin; propodus shorter than merus, robust setae along anterior margin; dactylus nodulate. *Pereopod 7* coxa subtriangular; basis oval shape; merus longer than wide, few robust setae; carpus longer than wide, robust setae along anterior margin and posterior margin; propodus longer than wide provided with several robust setae; dactylus nodulate.

####### Pleon

*Pleopods 1–3* peduncle shortened and slightly expanded distally; outer ramus longer than inner ramus; rami multiarticulate. *Uropod 1* peduncle with several robust setae lining at the medial margin and distally; rami subequal in length. *Uropod 2* peduncle provided with 2 robust setae and a robust seta distally; rami subequal in length. *Uropod 3* well developed; both rami with long plumose setae; outer ramus 2 biarticulate; inner ramus shorter than outer ramus. *Telson* about 5/6 cleft; both lobes with 2 setae apically and a seta medially.

**Figure 3b. F8:**
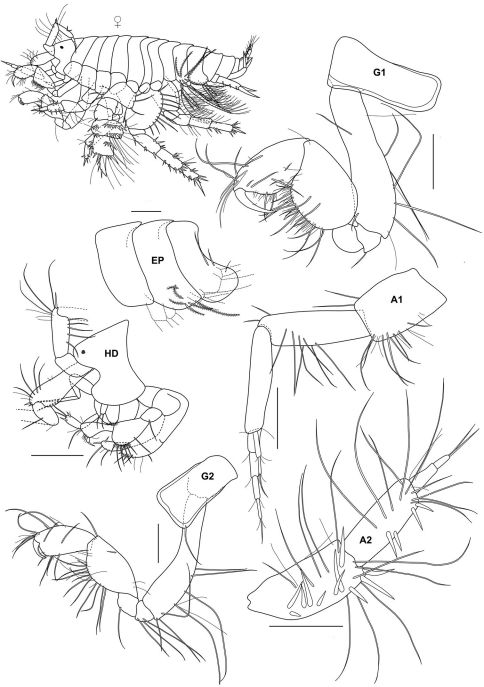
*Urothoe tinggiensis* sp. n., holotype, female, (UKMMZ-1399), 2.0 mm. Pulau Tinggi, Johor, South China Sea. All scales represent 0.1 mm.

**Figure 3c. F9:**
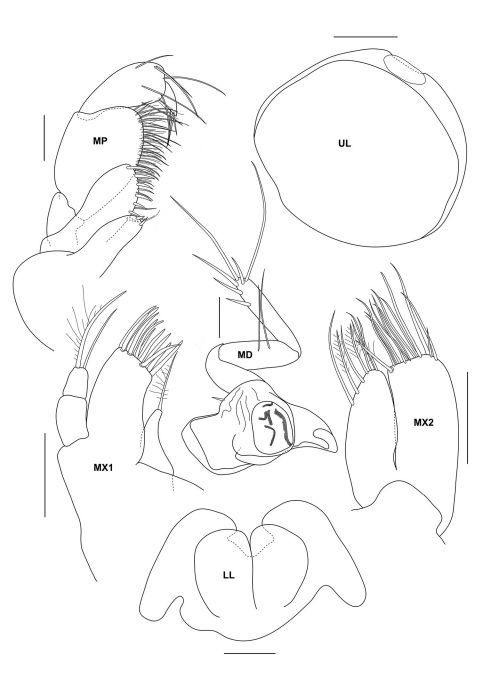
*Urothoe tinggiensis* sp. n., holotype, female, (UKMMZ-1399), 2.0 mm. Pulau Tinggi, Johor, South China Sea. All scales represent 0.05 mm.

**Figure 3d. F10:**
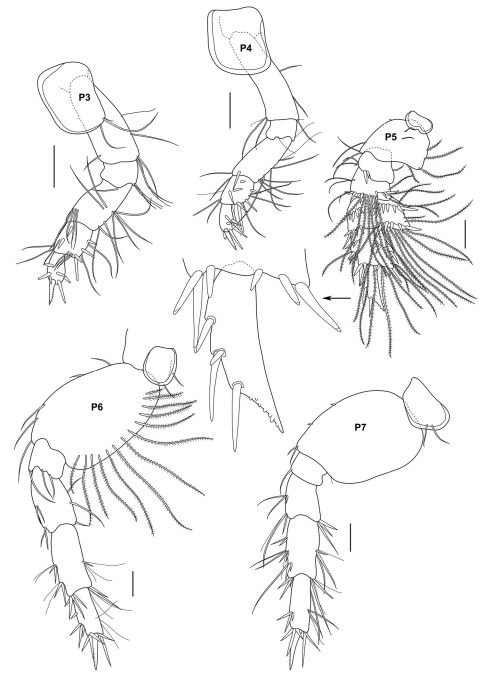
*Urothoe tinggiensis* sp. n., holotype, female, (UKMMZ-1399), 2.0 mm. Pulau Tinggi, Johor, South China Sea. All scales represent 0.1 mm.

**Figure 3e.**
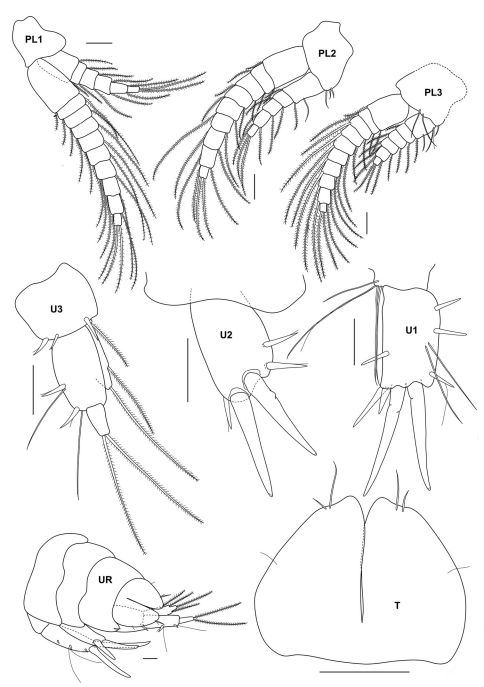
*Urothoe tinggiensis* sp. n., holotype, female, (UKMMZ-1399), 2.0 mm. Pulau Tinggi, Johor, South China Sea. All scales represent 0.05 mm.

###### Remarks.

According to the proposed classification of the ‘*falcata*’ group by Barnard (1962), members of this group possess dissimilar gnathopods; gnathopod 1 simple, elongated and slender propodus, bearing no palm; gnathopod 2 has a suboval or slightly expanding propodus with a distinct, rounded palm. With these characters our species is clearly attributed to the ‘*falcata’* group. However, there are perceivable differences that can be ruled out between the new species and all the members of the group. As such, the setose dactylus of pereopod 5 in *Urothoe tinggiensis* is readily distinguished from the non-spinose dactylus of pereopod 5 in *Urothoe pinnata* and *Urothoe tumorosa*. Differing from *Urothoe platydactyla* in the expanding and elliptical shape of the pereopod 5 dactylus. Both the pereopod 6 and 7 are somewhat different from *Urothoe platypoda*; the merus and carpus in *Urothoe tinggiensis* is considerably linear and elongated while the merus and carpus of both pereopod 6 and 7 in *Urothoe platypoda* are lobed posteriorly. The enormously produced coxa 4 in *Urothoe coxalis* is contrastingly distinctive from the normal subquadrate coxa 4 of *Urothoe tinggiensis*. The new species appears to have close affinities to *Urothoe orientalis* due to the wide carpus of pereopod 5 but clearly distinguished from the latter in the shape of propodus of gnathopod 1 and the absence of robust setae on the outer ramus of uropods 1 and 2. *Urothoe tinggiensis* is also distinguished from *Urothoe oniscoides* by the presence of eyes. The new species has some remarkable similarities with *Urothoe cuspis* Imbach, 1969 from Bay of Nhatrang, Vietnam, but seem to be different from the latter in the acuminate cuspidate coxa 2.

###### Distribution.

*Malaysia*. Johor: Pulau Tinggi.

## Supplementary Material

XML Treatment for Urothoe
